# KLHL14: A Novel Prognostic Biomarker and Therapeutic Target for Ovarian Cancer

**DOI:** 10.1155/2022/9799346

**Published:** 2022-08-22

**Authors:** Xingwei Wang, Ru Sun, Xia Hong, Chen Chen, Yan Ding

**Affiliations:** ^1^Department of Obstetrics and Gynecology, The Affiliated Hospital of Yangzhou University, Yangzhou University, Yangzhou 225001, China; ^2^Jiangsu Cancer Hospital and Jiangsu Institute of Cancer Research and the Affiliated Cancer Hospital of Nanjing Medical University, Nanjing 210008, China; ^3^The Comprehensive Cancer Centre of Nanjing Drum Tower Hospital, The Affiliated Hospital of Nanjing University Medical School and Clinical Cancer Institute of Nanjing University, Nanjing 210008, China

## Abstract

Ovarian cancer (OV) is a gynaecological malignancy that poses a serious risk to the health status of women. To date, effective molecular markers are unavailable for the diagnosis and management of ovarian malignancies. In this study, we aimed to investigate the molecular markers associated with the development of this cancer. We used bioinformatic analysis to determine the molecules and genes related to ovarian cancer using the gene expression profiling interactive analysis (GEPIA) and the cancer genome Atlas (TCGA) databases. In addition, we examined the genes and mechanisms underlying ovarian cancer. Our results showed that the KLHL14 gene is overexpressed in some cancers. An increase in the KLHL14 expression indicates a poor prognosis in patients with ovarian cancer. Results of immune cell infiltration analysis and half-maximal inhibitory concentration (IC50) analysis provide novel insights into the treatment of ovarian cancer. KLHL14 is anticipated to emerge as a novel molecular marker specifically for ovarian carcinoma.

## 1. Introduction

Ovarian cancer is among the most lethal gynaecological malignancies [1]. It involves a heterogeneous group of malignancies that differ in aetiology, molecular biology, and many other characteristics [2, 3]. Approximately, 90% of ovarian cancers are of epithelial origin, and the prevalent subtype of epithelial ovarian cancer is serous carcinoma [4]. Although several patients demonstrate no evidence of disease following standard treatment, nearly 70% of patients report relapse within 3 years of therapy [5]. Patients are diagnosed at the most advanced stages of the disease with stages III and IV accounting for 51% and 29%, respectively. From 2007 to 2013, the 5-year overall survival of patients with stage III and stage IV ovarian cancer was 42% and 26%, respectively [6]. The poor outcomes in these patients may be attributed to the advanced stage at the time of diagnosis, a high incidence of relapse and the development of resistance to treatments.

With the improvements in radical surgery as well as chemotherapy-based therapeutic management strategies in epithelial ovarian cancer, the 5-year overall survival continues to be 40% for advanced ovarian cancer. Therefore, developing novel treatment options is critical. Molecular-targeted treatments offer hope for precision management of ovarian cancer with greater specificity and a lower level of toxicity. Therefore, active and tolerable new targeted agents are required to enhance the prognosis of these patients.

KLHL14 belongs to the Kelch-like gene family. The Kelch-like gene family contains a BTB/POZ domain, a BACK domain and five to six Kelch motifs [7]. Previous studies indicated an association between KLHL genes and cancer progression; however, the mechanism underlying the role of KLHL genes remains to be established. Thus, further studies are required to investigate the role of KLHL14.

Bioinformatic analysis is an advanced approach widely used in cancer-related investigations, and several studies have been performed using this technique to identify cancerous genes. Although substantial progress has been made in understanding of the biology of ovarian cancer, novel biomarkers need to be identified for developing targeted therapy for ovarian cancer patients [8]. RNA-sequencing-based investigations have explored the function of certain molecules and genes involved in the pathogenesis and recurrence of ovarian cancer.

The Cancer Genome Atlas (TCGA) and the Gene Expression Profiling Interactive Analysis (GEPIA) databases were used to determine the function of KLHL14 in anticipating overall survival (OS). Our results showed that an increased expression level of KLHL14 was a predictor of poor OS. Additional analysis of the mechanisms underlying the association between high expression levels of KLHL14 and ovarian cancer were conducted using pathway analysis, immune cell infiltration analysis, functional analysis, and half-maximal inhibitory concentration (IC50) analysis. Overall, our results indicated that KLHL14 can function as a biomarker for ovarian cancer and provide novel insights into the mechanisms underlying the microenvironment of serous ovarian cancer.

## 2. Material and Methods

### 2.1. Transcriptional Expression of Uncoupling Proteins

The GEPIA is an interactive web application tool that is instrumental in gene expression analysis. It contains 8587 normal samples and 9736 cancerous samples. Comparisons of KLHL14 expression between cancerous tissues and normal tissues were performed using the fragments per kilobase of transcript per million mapped reads (FPKM) of mRNA in the GEPIA database (https://gepia.cancer-pku.cn/) [9, 10].

### 2.2. TCGA Data

The GTEx dataset (https://commonfund.nih.gov/GTEx) contained an RNA-Seq dataset of 88 normal ovarian tissues. TCGA (https://gdc.nci.nih.gov/) provided transcriptome data as well as clinical data for 379 patients with ovarian cancer. All information are included in Table 1.

### 2.3. Survival Analysis

For survival analyses, we used the ovarian cancer gene expression profiles of ovarian cancer tissue samples from the TCGA-OV cohort. Additionally, the Kaplan–Meier (KM) plotter (https://www.kmplot.com/) was used to collect the data of OS using the mRNA expression of candidate target genes.

### 2.4. Identification of Differentially Expressed Genes

We identified differentially expressed genes (DEGs) between the high- and low-expression groups in ovarian cancers [11]. The thresholds were *p* < 0.05 and |log2 foldchange| > 1 [12].

### 2.5. Functional Analysis

To further verify the fundamental role of the potential targets, we used functional data enrichment to analyse the data. Gene ontology (GO) is an extensively used method for annotating genes with roles, particularly molecular function (MF), cellular components (CC), and biological pathways (BP). The Kyoto Encyclopedia of Genes and Genomes (KEGG) enrichment analysis is a practical resource for examining the roles of genes and related high-level functional information of the genome. To thoroughly comprehend the role of mRNA in carcinogenesis, we used the ClusterProfiler package (version: 3.18.0) in R to evaluate the GO function of possible targets and enrich the KEGG pathway. The ggplot2 package in the R software was used to create a boxplot, whereas the p heatmap package in the R software was used to generate a heatmap [13].

### 2.6. Mutation Analysis

The data pertaining to mutations were retrieved and visualised using the maftools package in the R software. The histogram showed genes with a higher mutational frequency of KLHL14 [14, 15].

### 2.7. Immune Cell Infiltration Analysis

RNA-sequencing expression profiles and related clinical information for KLHL14 were retrieved from the TCGA dataset (https://portal.gdc.com) [16]. To determine the reliability of the immune score evaluation findings, the immunedeconv program in the R package was used, which includes six of the recent algorithms, such as EPIC, CIBERSORT, quanTIseq, and xCell. These algorithms have been tested, and each has a distinct advantage [17–20].

### 2.8. IC_50_ Analysis

We used the largest publicly accessible pharmacogenomics database (the Genomics of Drug Sensitivity in Cancer (GDSC), https://www.cancerrxgene.org/) to anticipate the chemotherapeutic response for every sample. The anticipation process was successfully executed using the R package pRRophetic. The IC_50_ values of the samples were derived using ridge regression. All parameters were set to default values, with the batch impact of combat as well as type of tissues, and the duplicate gene expression was presented as a mean value [21–23].

### 2.9. Human Protein Atlas Analysis

The Human Protein Atlas (HPA) is a database that used immunohistochemical methods to perform expression analysis of different proteins in normal and cancer tissues. Images of immunohistochemical staining of tissues obtained from ovarian cancer patients were retrieved from the online HPA database.

## 3. Results

### 3.1. Pan-Cancer Analysis of KLHL14

**Figure 1:**
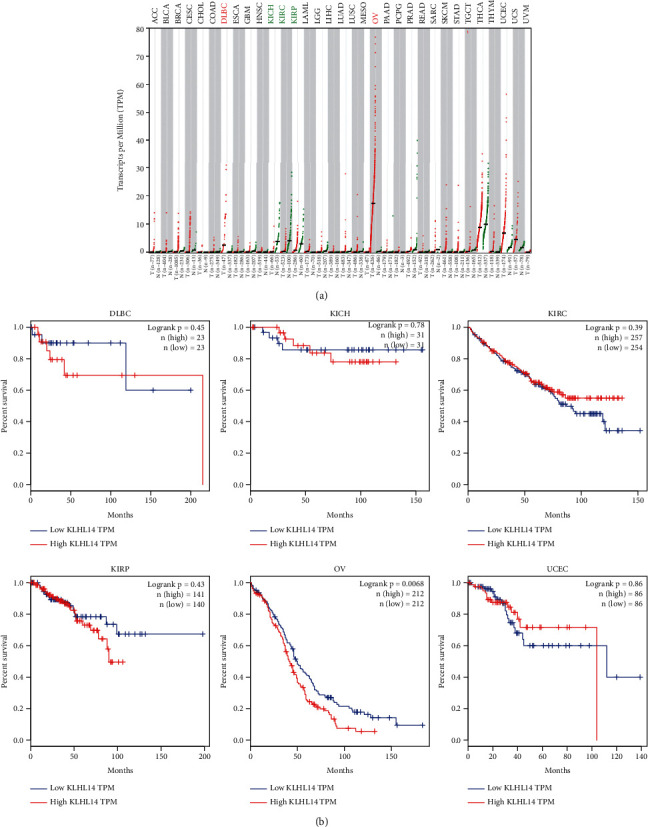
(a) Gene expression profiling interactive analysis (GEPIA) data showing the expression levels of human KLHL14 in different types of cancer. (b) Overall survival (OS) in groups showing high and low expression levels of KLHL14 in the dysregulated cancers.

Of the 33 cancer types analysed, lymphoid neoplasm diffuse large B-cell lymphoma (DLBC), kidney chromophobe (KICH), kidney renal clear cell carcinoma (KIRC), kidney renal papillary cell carcinoma (KIRP), ovarian serous cystadenocarcinoma (OV), and uterine corpus endometrial carcinoma (UCEC) showed higher expression levels of KLHL14, demonstrating that KLHL14 plays an important role in multiple cancer types (Figure 1(a)). The KM OS plots of KLHL14 in OV are shown in Figure 1(b).

### 3.2. Validating the Expression and Prognostic Relevance of KLHL14 in Other Datasets

**Figure 2:**
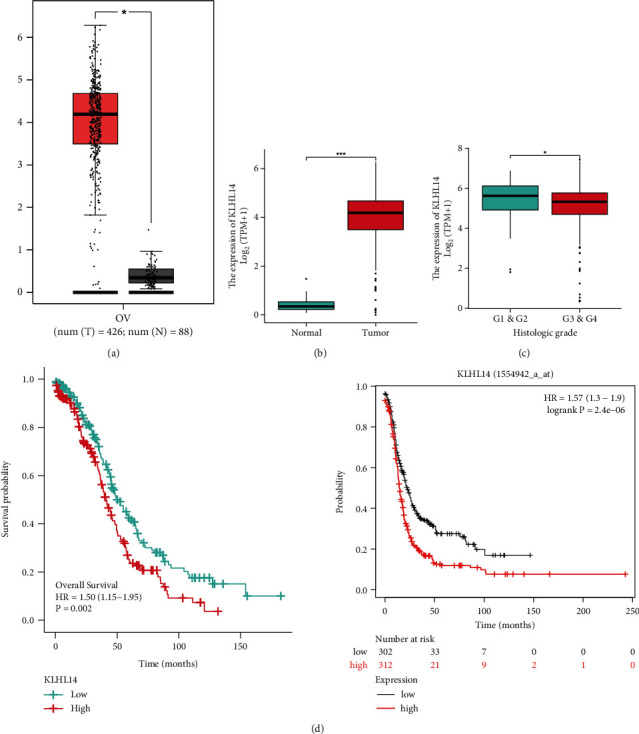
(a) The boxplot of KLHL14 expression in ovarian cancer using the gene expression profiling interactive analysis (GEPIA) database. (b) The KLHL14 expression level in cancer and normal tissues in TCGA database. (c) Association between KLHL14 and the pathological stages of ovarian cancer. (d) The Kaplan–Meier (KM) curves revealed overall survival (OS) in ovarian cancer using the Cancer Genome Atlas (TCGA) and KM database.

The boxplot from the GEPIA database showed that KLHL14 was dysregulated in the OV samples (Figure 2(a)). To further explore the clinical relevance of KLHL14 in OV, we used data from TCGA database. The findings affirmed that the KLHL14 was significantly upregulated in the OV samples (Figure 2(b)). The expression of KLHL14 significantly increased with an increase in the pathological grade of OV (Figure 2(c)).

### 3.3. Prognostic Potential of KLHL14 in OV

The KM plot based on TCGA database and the KM database revealed that a high KLHL14 expression was linked to a poorer prognosis among individuals with OV (Figure 2(d)).

### 3.4. Identification of DEGs

**Figure 3:**
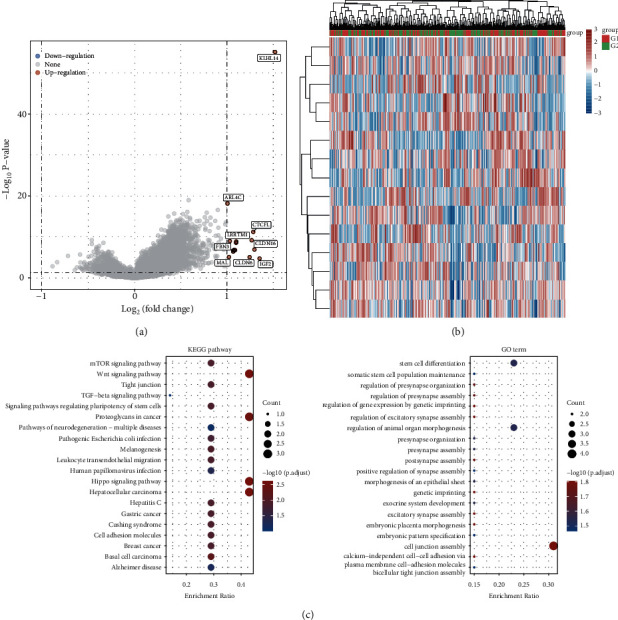
(a) Volcano plot: the volcano plot was generated using the fold change values and P-adjust. Red dots indicate upregulated genes, blue dots indicate downregulated genes, and grey dots indicate not significant. (b) The heatmap of the differential gene expression, where different colours illustrate the trend of gene expression in different tissues. (c) Functional enrichment: The enriched Kyoto Encyclopedia of Genes and Genomes (KEGG) signalling pathways and Gene Ontology (GO) terms.

TCGA data were used to standardise data and transform raw data into expression values. The significance analysis based on the limma package was applied to detect the DEGs between tissue samples showing high and low expression levels of KLHL14. *p* value <0.05 and |logFC| > 1 was used as the cut-off criteria to guide the selection of significant DEGs (Figures 3(a) and 3(b)).

### 3.5. Enrichment Analyses

To ascertain the biological roles of the DEGs, we performed functional and pathway enrichment analyses. The findings of the GO analysis affirmed that upregulated and downregulated DEGs were substantially enriched in functions such as development of the uterus. These findings were consistent with those of the KEGG pathway in that the KLHL14 may affect OV via pathways like the Wnt signalling pathway (Figure 3(c)).

### 3.6. Mutation and Copy Number Variation Analysis

**Figure 4:**
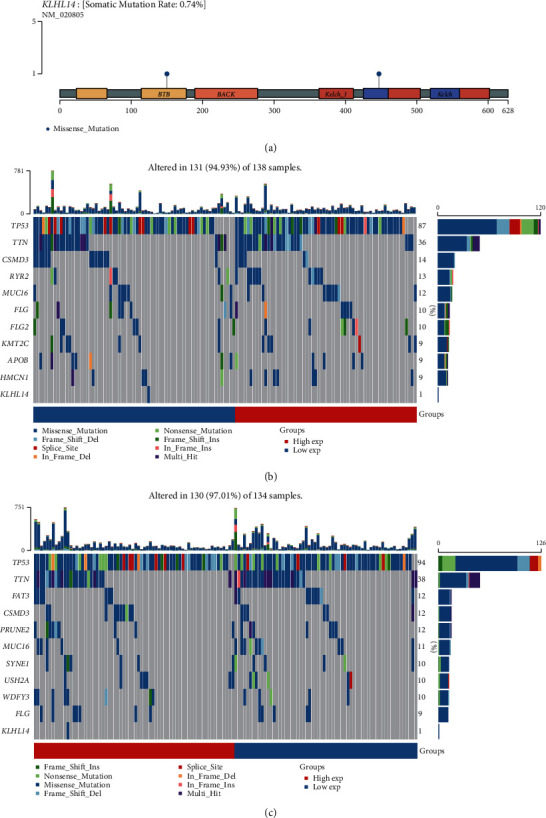
(a) Lollipop charts of the mutated KLHL14 gene, the somatic mutation rate and the name of somatic mutation. (b–c) Oncoplot shows the somatic landscape of the OV cohort ((b) low expression group, (c) high expression group). Genes and samples were ordered based on their mutation frequencies and disease histology, respectively, as shown by the annotation bar.

Data regarding mutations was plotted using the mutation sites of KLHL14 (Figure 4(a)). Patients with low expression levels of KLHL14 showed remarkable mutations in the TP53, TTN, CSMD3, RYR2, and MUC16 genes, corresponding to 87%, 36%, 14%, 13%, and 12%, respectively. However, patients with high expression levels of KLHL14 showed substantial mutations in the TP53, TTN, FAT3, CSMD3, and PRUNE2 genes, corresponding to 94%, 38%, 12%, 12%, and 11%, respectively (Figures 4(b)-4(c)).

### 3.7. Impact of KLHL14 Gene on Immune Cell Infiltration in TCGA-OV Patients

**Figure 5:**
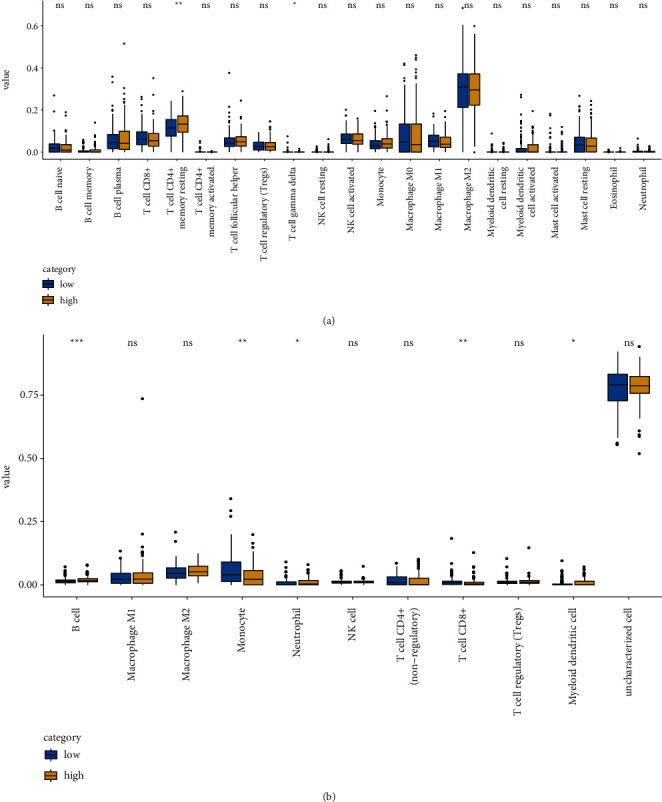
Immune cell score heatmap, where different colours indicate different distribution of expression in various samples (^*∗*^*p* < 0.05, ^∗∗^*p* < 0.01,^∗∗∗^*p* < 0.001; where asterisks (^*∗*^) indicate significance levels). (a) CIBERSORT. (b) EPIC.

**Figure 6:**
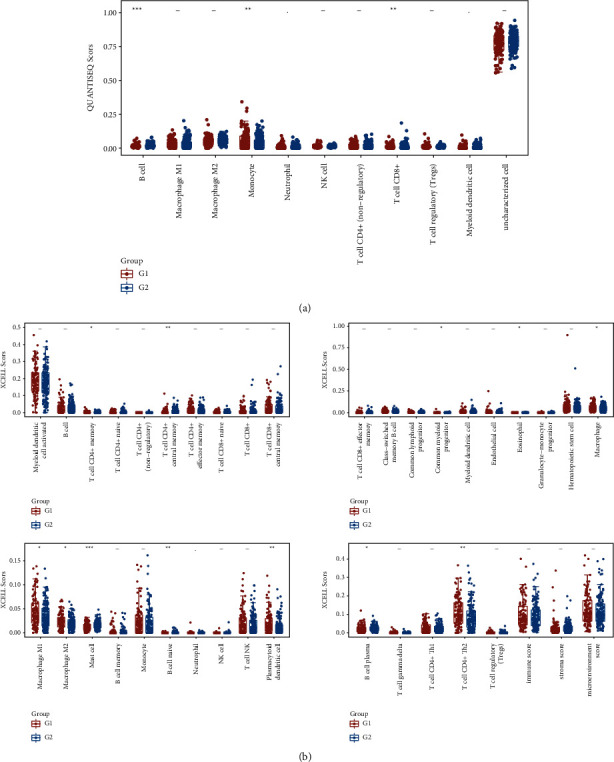
Immune cell score heatmap, with different colours indicating the varying distribution of expression in various samples (^*∗*^*p* < 0.05, ^∗∗^*p* < 0.01, ^∗∗∗^*p* < 0.001; where asterisks (^*∗*^) indicate significance levels). (a) quanTIseq. (b) xCell.

To investigate the association between KLHL14 gene expression and immune cell infiltration in the microenvironment of OV, immune cell invasion in the tumour microenvironment (TME) was assessed using the EPIC, CIBERSORT, quanTIseq, and xCell algorithms. The landscape of immune cell infiltration in TCGA-OV TME and the results of the correlation analysis of the immune cell scores are shown in Figures 5 and 6. KLHL14 was verified to be substantially positively correlated with T cells.

### 3.8. Response to Chemotherapy Drugs

**Figure 7:**
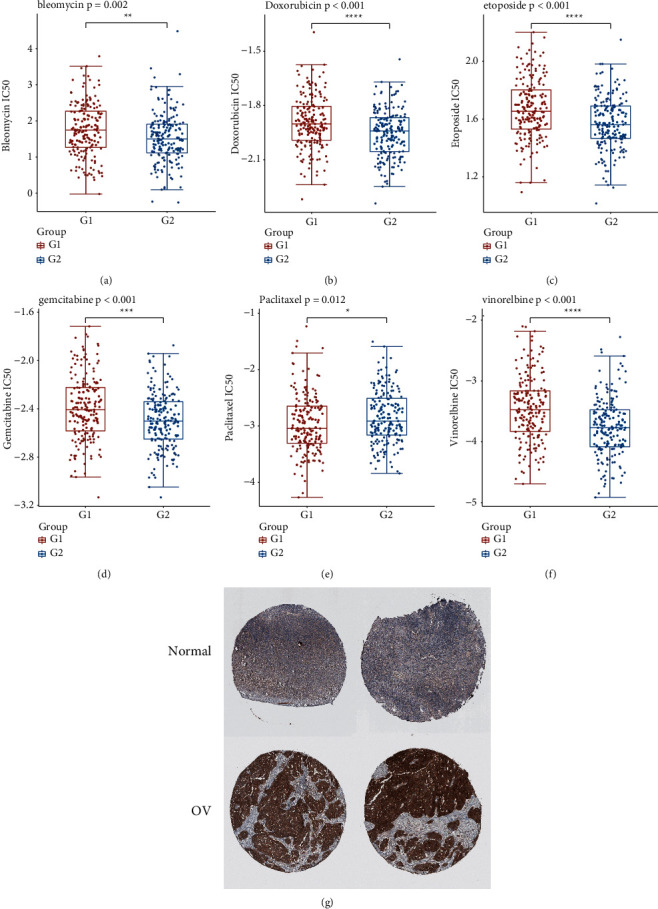
(a–f) Distribution of the half-maximal inhibitory concentration (IC50) score. The abscissa denotes various groups of samples and the ordinate stands for the distribution of the IC50 score. Different colours indicate different groups, and the top left denotes the significance *p*-value test method (^*∗*^*p* < 0.05, ^∗∗^*p* < 0.01, ^∗∗∗^*p* < 0.001; where asterisks (^*∗*^) indicate significance levels). (g) The results of immunohistochemical (IHC) staining of KLHL14 expression using the Human Protein Atlas (HPA) database.

We used the GDSC database to analyse the IC_50_ value in the groups showing high- and low expression levels of KLHL14 groups; our results showed that individuals with low expression levels of KLHL14 were responsive to the antitumour drugs like bleomycin, doxorubicin, etoposide, gemcitabine and vinorelbine, and patients showing low expression levels of KLHL14 can take paclitaxel (Figures 7(a)-7(f)).

### 3.9. Validation of KLHL14 Expression via the HPA Database

The protein expression of KLHL19 was analysed using immunohistochemical staining images obtained from HPA. KLHL19 protein showed a higher expression in the OV tissue than in the normal tissue (Figure 7(g)).

## 4. Discussion

Ovarian cancer is one of the most frequent gynaecologic malignancies worldwide [24]. Over the past decades, considerable efforts have been undertaken to identify accurate biomarkers for the detection of ovarian cancer. For example, a series of genes, including LAMP3, MAGE-A9, and LAMP1, have been identified thus far [25–28]. However, the prognosis of patients with ovarian cancer remains dismal. The 5-year survival rate of patients with advanced-stage ovarian cancer continues to be <30%. To the best of our knowledge, this is the first study in which overexpression of KLHL14 has been reported specifically in ovarian cancer. We analysed KLHL14 expression levels in different types of human cancers, and we observed that KLHL14 was significantly upregulated in ovarian cancer compared to that in other kinds of human cancers. Moreover, compared with normal tissues, OV tissues obtained from patients in all stages of ovarian cancer showed the KLHL14 overexpression. These results strongly suggested that KLHL14 could act as an early diagnostic biomarker for OV.

With advancements in the technology of radiotherapy and a corresponding decrease in the toxicities, radiotherapy has been widely used in the treatment of recurrent OV [29]. However, to date, the outcome of patients with OV has not improved [30]. The mechanism underlying the development of resistance to chemotherapeutic agents in ovarian cancer is very complex [31]. Chemosensitivity and drug resistance analyses are essential for selecting appropriate chemotherapeutic regimens and in survival assessments for patients with OV [31]. Therefore, novel approaches to restoring the chemotherapy sensitivity of ovarian cancer are urgently required. The expression of KLHL14 can provide novel insights for better management of patients with OV.

Previous studies have indicated that KLHL genes are associated with cancer. For example, KLHL19 can inhibit the proliferation of lung cancer by decreasing the transcriptional activity of NRF2 [32]. A recent study showed that Kelch-like protein 14 promoted the development of B-1a cells but inhibited the development of B-1b cells. Moreover, Wu et al. found that KLHL14 was hypomethylated in endometrial cancer [33]. KLHL14 is overexpressed in OV; however, the mechanisms underlying this overexpression remain unclear [34]. In this study, KLHL14 protein was overexpressed in OV samples. Dysregulation of KLHL14 may be related to pathways such as the Wnt signalling pathway. Many malignancies, including ovarian and endometrial cancer, show an abnormal expression of the Wnt signalling receptor ROR1. Additionally, KLHL14 may affect the outcome of patients with OV owing to changes in TME. Cancer progression is influenced by immune cells. Immunotherapies for ovarian cancer are currently in the early phases of development. However, *in vitro* and *in vivo* studies have examined a growing number of therapeutic immunotherapies.

We categorised cancerous samples into high- and low-KLHL14-expression groups to further explore the KLHL14-related genes and molecular characteristics. Analysis of the mutation characteristics of the two KLHL14 expression groups indicated remarkable variations in the mutations between the two groups. The group with low KLHL14 expression levels showed frequent mutations in the TP53, TTN, and FAT3 genes. The analysis of IC50 showed that antitumour drugs, including bleomycin, doxorubicin, etoposide, gemcitabine, and vinorelbine, were more suitable for patients with a high expression of KLHL 14.

## 5. Conclusion

To the best of our knowledge, this is the first study in which KLHL14 has been proposed as an early diagnostic target and a predictor of the poor prognosis of OV. Significantly high expression levels of KLHL14 were observed in all stages of OV. KM survival curve analysis demonstrated that overexpression of KLHL14 was correlated with a significantly shorter OS in patients with OV. Moreover, bioinformatic analyses indicated that KLHL14 played an important role in the microenvironment and drug sensitivity [35].

## Figures and Tables

**Table 1:** Baseline values of KLHL14 expression and clinical information.

Characteristic	Low expression of KLHL14	High expression of KLHL14	*p*
*n*	189	190	
FIGO stage, *n* (%)			0.777
Stage I	0 (0%)	1 (0.3%)	
Stage II	10 (2.7%)	13 (3.5%)	
Stage III	147 (39.1%)	148 (39.4%)	
Stage IV	30 (8%)	27 (7.2%)	
Primary therapy outcome, *n* (%)			0.553
PD	10 (3.2%)	17 (5.5%)	
SD	12 (3.9%)	10 (3.2%)	
PR	22 (7.1%)	21 (6.8%)	
CR	110 (35.7%)	106 (34.4%)	
Race, *n* (%)			0.310
Asian	8 (2.2%)	4 (1.1%)	
Black or African American	10 (2.7%)	15 (4.1%)	
White	165 (45.2%)	163 (44.7%)	
Age, years, median (IQR)	57 (49, 68)	60 (53, 68)	0.091

## Data Availability

The datasets used and/or analysed during the current study are available from the corresponding author on reasonable request.
